# Book Reviews

**Published:** 1799-03

**Authors:** 


### CHEMISTRY AND NATURAL PHILOSOPHY.

*Demonſtration de la fauſſetté,* &c. "*A Demonſtration of the Falſity of the Principles adopted by the Modern Chemiſts ; being a Supplement to the Treatiſe on the Diſſolution of Metals ;*" by Citizen MONNET. 8vo. 400 pp. (4 Liv. 10 ſols.) *Paris.* Janſen.

THE author begins with combating the opinion of Scheele, reſpecting the *ſpathic acid,* which he calls the *vitreous acid of Spath.* He next adverts to the *ſuppoſed ſaccharine acid* which, in his opinion, is no more the acid of ſugar, than the vitriolic acid diſtilled upon ſpath is the acid of that ſubſtance ; an opinion in which he is ſupported by the authority of MACQUER and WIEGLEB.—The acid of tartar, according to citizen Macquer, is compoſed of marine accid, oil, earth, and fixed alcali.—In examining the properties of galls, and the *gallic acid,* he attempts to refute MORVEAU's aſſertion, that the colouring principle (aſtringent matter) contained in galls, affords this particular acid.

Although the author does not fail to illuſtrate his ſingular aſſertions by numerous experiments, inſtituted by himſelf, on the chemical properties of the ſubſtances he has analyzed, yet we are perſuaded that his illuſtrations would have ſuffered no derogation from the merit they poſſeſs, had he treated his reſpectable opponents with more liberality and politeneſs ; for we frequently meet with ſuch expreſſions in his polemical treatiſe, as are equally unworthy of a philoſopher and a man of ſcience.

*Grundriſs der Naturlehre,* &c. *Elements of Natural Philoſophy* ; being a Text-book for the Uſe of Students attending his Lectures : By D. L. BOURGUET, Prof. of Chemiſtry in the Royal Medico-Chirurgical College of Berlin. With two Plates. 8vo. 326 pp. *Berlin.* Hartmann. 1798.

THE preſent ſummary was compiled, in conſequence of an intimation given to the author, by the committee of the Pruſſian government appointed to ſuperintend and regulate the affairs of literary inſtitutions ; as there was evidently no elementary treatiſe in the German language, in which the outlines of phyſical ſcience were properly connected with the fundamental principles of modern chemiſtry. This intention the author has accurately and completely fulfilled ; and, although he is conſiderably indebted to the labours of his predeceſſors, who have furniſhed him with his principal materials in general phyſics, as well as in chemiſtry, particularly to *Klügel* and *Gren,* he is nevertheleſs entitled to well-earned commendation for having judiciouſly digeſted a compendious and valuable ſchool-book.

*An Inaugural Diſſertation, ſhewing in what Manner peſtilential Vapours acquire their acid Quality, and how this is neutralized and deſtroyed by Alkalies,* &c. &c." By A. C. LENT, Citizen of the State of New-York. 8vo. 54 pp. *New-York.* Swords. (May 1798.)

THIS perſpicuous and well-written eſſay poſſeſſes a juſt claim to honourable mention here ; and the more ſo, becauſe the doctrines advanced in it, are profeſſedly drawn from the Lectures of the preſent Profeſſor of Chemiſtry, in Columbia-College, Dr. MITCHILL, the American Fourcroy.

We have not room to inſert a ſynoptical view of this new doctrine which, in a great meaſure correſponds with Dr. Trotter's opinion relative to to the extirpation of peſtilential vapours, and of which he has announced his intention to give an abſtract in the ſecond volume of his 'Medicina Nautica*.'

*A Sketch of the Revolutions in Chemiſtry.* By T. P. SMITH. 8vo. 40 pp. *Philadelphia.* Smith. 1798.

THIS 'Sketch' compriſes the ſubſtance of an annual oration delivered before the Chemical Society of Philadelphia, relative to the progreſs of chemiſtry in different countries, within the preceding year. Mr. Smith has materially deviated from the plan preſcribed by the ſociety; for, in lieu of confining himſelf to the chemical hiſtory of the year, he preſents a general view of the revolutions, which this branch of knowledge has undergone in different ages, and countries.—Although we differ from the author in ſome of the poſitions advanced by him, for inſtance, that the ancient allegories which appear to have a *phyſical* relation, "muſt be paſſed over as the unmeaning offſpring of ignorance and ſuperſtition," we muſt ingenuouſly allow that this oration is not without a conſiderable ſhare of merit; as it contains an intereſting, though incomplete, hiſtorical account of a ſcience, in which a new æra is opened to the philoſophic inquirer.—We ſhall conclude this article by expreſſing our ſatiſfaction on learning, that the ſcience of chemiſtry begins to be ſtudied with taſte and avidity, by the females of the tranſatlantic continent.

* Vid. Under the head of '*Medical and Phyſical Intelligence*;' p. 80 of this number.

